# A Case Report on Left-sided Appendicitis with Intestinal Malrotation

**DOI:** 10.7759/cureus.6687

**Published:** 2020-01-17

**Authors:** Himal Kharel, Nishan B Pokhrel, Zeni Kharel, Dhruba Sah

**Affiliations:** 1 Orthopaedics, Tribhuvan University Institute of Medicine, Kathmandu, NPL; 2 Internal Medicine, Rochester General Hospital, Rochester, USA; 3 Surgery, Tribhuvan University Institute of Medicine, Kathmandu, NPL

**Keywords:** appendectomy, intestinal malrotation, left-sided appendicitis

## Abstract

Acute appendicitis is a mimicker of a wide range of gastrointestinal and genitourinary pathologies. The diagnosis becomes more challenging when it is associated with intestinal malrotation. A rare case of left-sided acute appendicitis with asymptomatic undiagnosed intestinal malrotation is reported. A 32-year-old male without known comorbidities presented with left-sided abdominal pain. Abdominal ultrasonography and computerized tomography scans showed intestinal malrotation with acute appendicitis. Exploratory laparotomy and appendectomy with Ladd’s band release via midline incision were performed, and the patient had no issues on follow-up. Given the rarity of acute appendicitis associated with intestinal malrotation, an increase in awareness of this anatomical variant is essential among emergency physicians, radiologists, and surgeons for prompt diagnosis and timely intervention.

## Introduction

Abdominal pain is one of the most common complaints for emergency visits. Acute appendicitis is the most common surgical cause of acute abdominal pain with overall lifetime risks for males and females being 8.6% and 6.7%, respectively [1]. The presentation of acute appendicitis mimics a wide range of gastrointestinal and genitourinary pathologies like gastritis, gastroenteritis, cholecystitis, pyelonephritis, and diverticulitis. Atypical presentation of acute appendicitis with a left lower quadrant pain could be misleading. As diverticulitis is most often considered as the differential diagnosis in patients with left-sided abdominal pain, it creates a diagnostic dilemma. Moreover, researchers have noticed an increasing prevalence of diverticulitis in younger adults than previously recognized [2].

Left lower quadrant pain caused by acute appendicitis can be due to a right-sided long appendix projecting into the left lower quadrant area and an abnormal left-sided location of the appendix [3]. The abnormal left-sided location of the appendix results from two congenital anomalies: intestinal malrotation and situs inversus totalis [4].

We report a case presenting with progressive and constant pain in his left lower abdomen which we presumed to be due to diverticulitis. However, abdominal ultrasonography (USG) and computerized tomography (CT) scans revealed left-sided acute appendicitis with intestinal malrotation, which significantly changed clinical management. He did well postoperatively and was discharged after a successful recovery.

## Case presentation

A 32-year-old gentleman without known comorbidities presented to the emergency department of Tribhuvan University Teaching Hospital, Maharajgunj, with complaints of insidious, progressive, constant pain in his left iliac fossa without radiation for two days. It was accompanied by nausea, vomiting, and anorexia. There was no history of hematochezia, melena, or fever, and his bowel and bladder habits were normal. He had no prior history of surgeries, drug abuses, or drug allergies. The patient was initially evaluated in a previous center for the same complaint where he was suspected as having diverticulitis.

**Figure 1:**
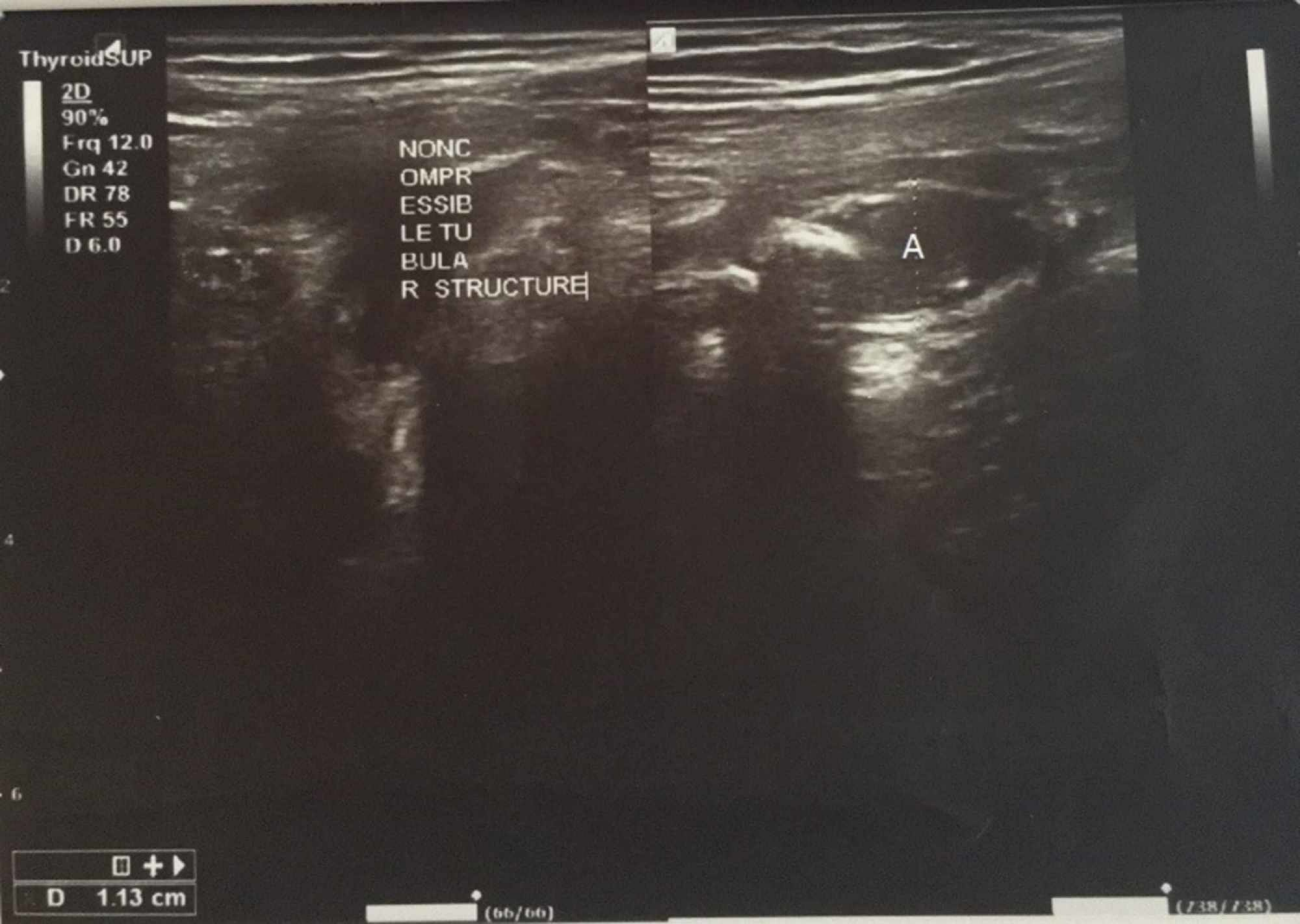
Ultrasonography of the abdomen showing a noncompressible blind-ended tubular structure without periappendiceal fluid (A)

**Figure 2:**
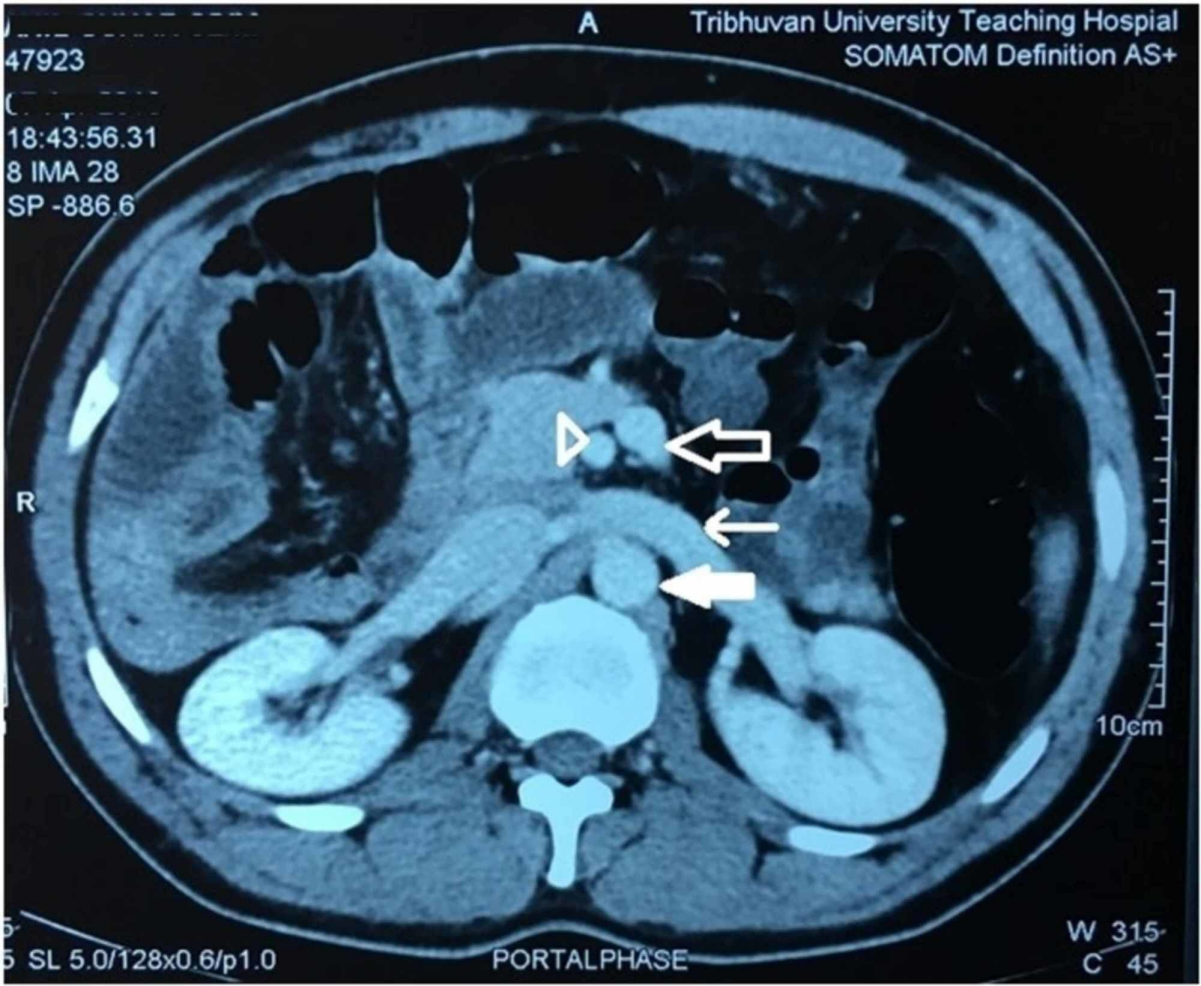
Axial CECT of the abdomen (portal phase) showing the inverse relationship between SMA (arrowhead) and SMV (unfilled block arrow) along with the presence of renal vein (line arrow) and absence of duodenum between SMA and aorta (filled block arrow) CECT, contrast-enhanced computed tomography; SMA, superior mesenteric artery; SMV, superior mesenteric vein

**Figure 3:**
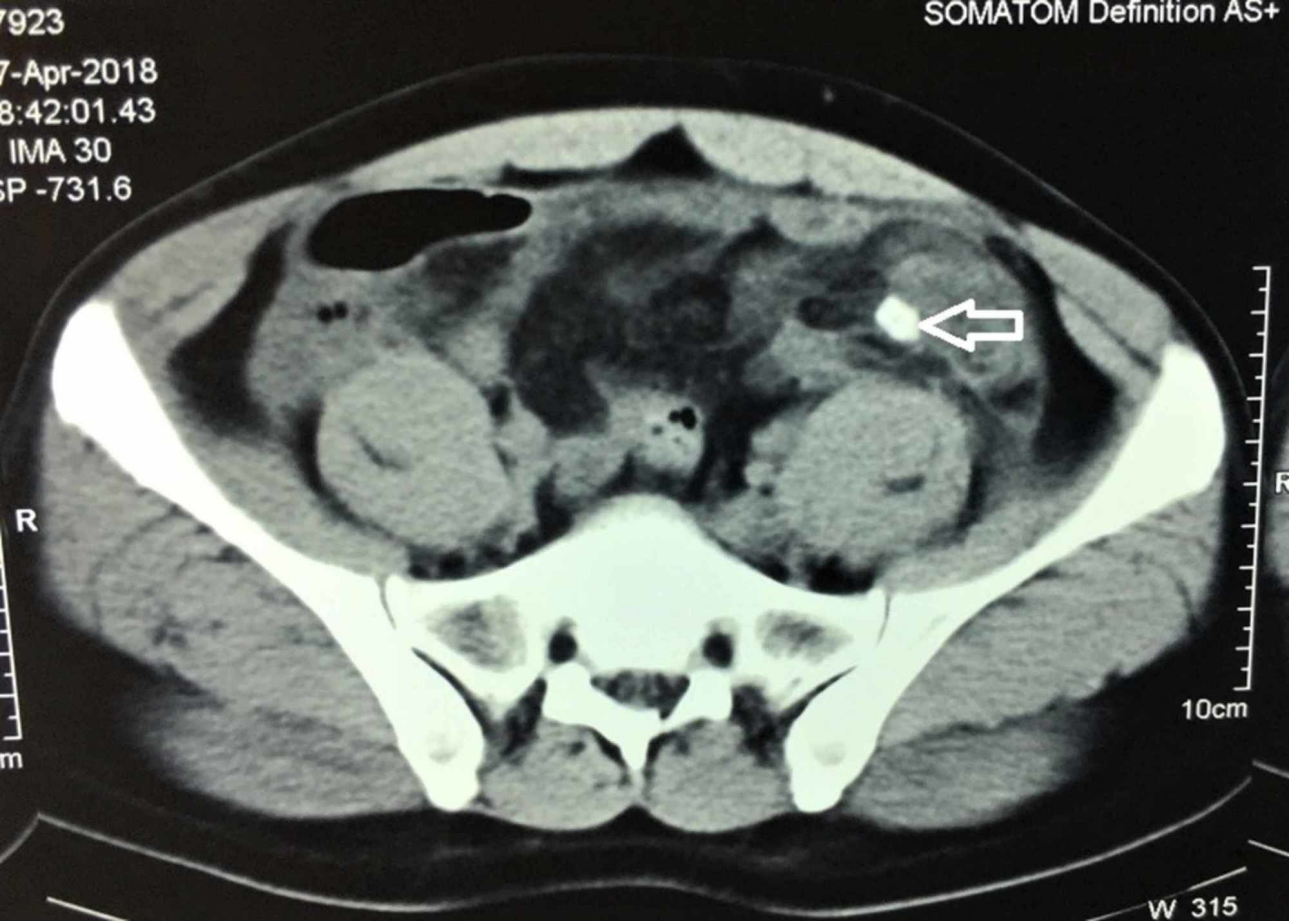
Axial noncontrast computerized tomography (NCCT) of abdomen and pelvis showing an appendicolith (unfilled block arrow)

**Figure 4:**
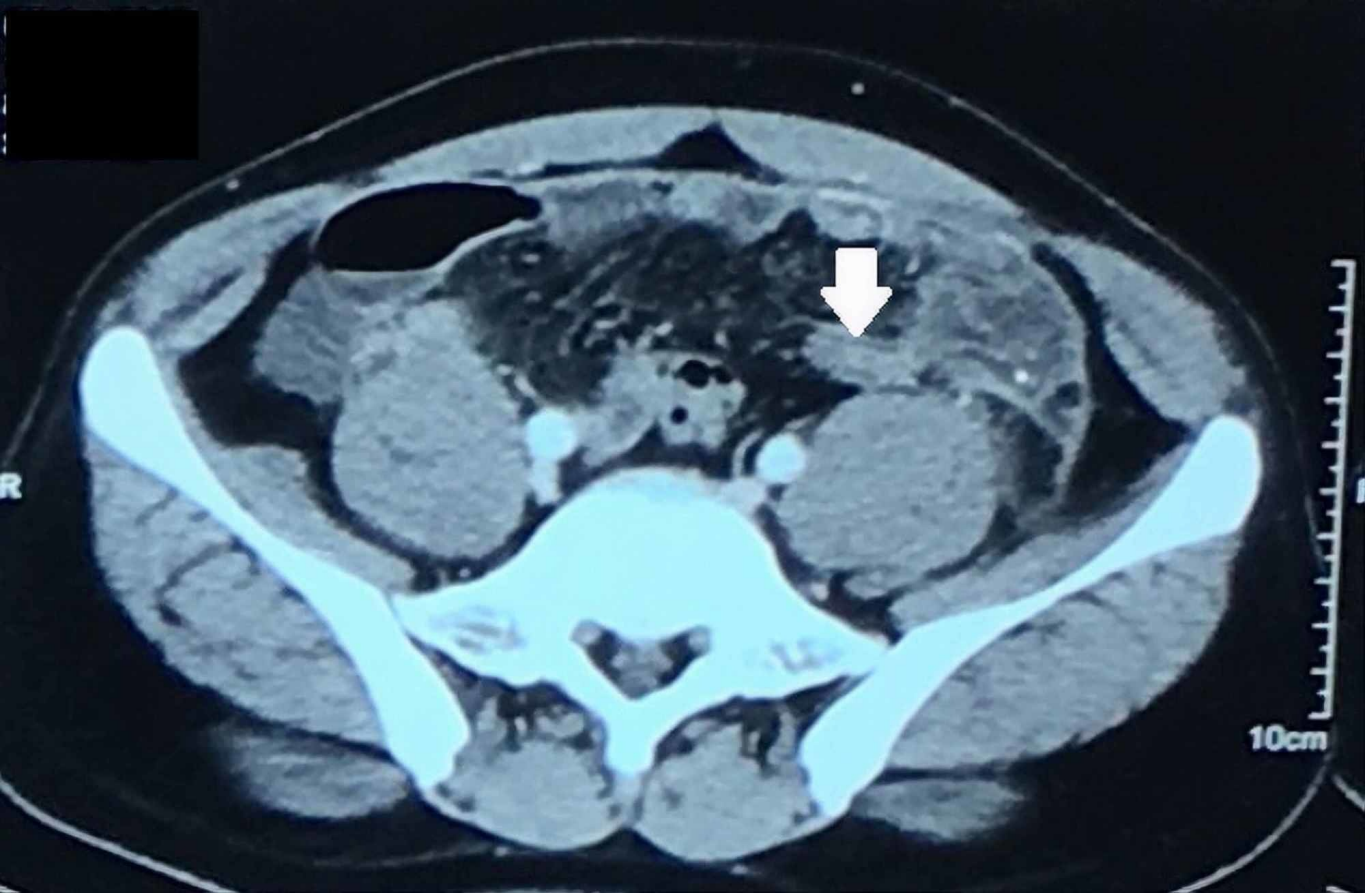
Axial CECT of abdomen and pelvis showing appendix (solid block arrow) arising from the cecum on left iliac fossa with thickened wall and periappendicular fat stranding CECT, contrast-enhanced computed tomography

On examination, the patient was found to be tachycardic. Abdominal examination revealed tenderness, rebound tenderness, guarding in left iliac fossa, and normal digital rectal examination. With the presumptive diagnosis of diverticulitis, USG of the abdomen and pelvis was performed to confirm our suspicion. However, we found a noncompressible, blind-ending, tubular structure with surrounding hyperechoic mesentery with probe tenderness in left iliac fossa without free fluid which we suspected to be left-sided acute appendicitis (Figure 1). Abdominal contrast-enhanced CT (CECT) scan showed a large bowel predominantly on the left side and small bowel predominantly on the right side. The relation between a superior mesenteric artery (SMA) and superior mesenteric vein (SMV) was inverted with SMA on the right side and SMV on the left side. Duodenum was absent between the SMA and aorta and was entirely on the right side (Figure 2). Appendix arising from cecum was noted in the left iliac fossa with thickened wall and periappendiceal fat stranding with two appendicoliths of 9×7 and 6×5 mm^2^ sizes (Figures 3, 4). Thus, the diagnosis of acute appendicitis with complete nonrotation of midgut was made. 

**Figure 5:**
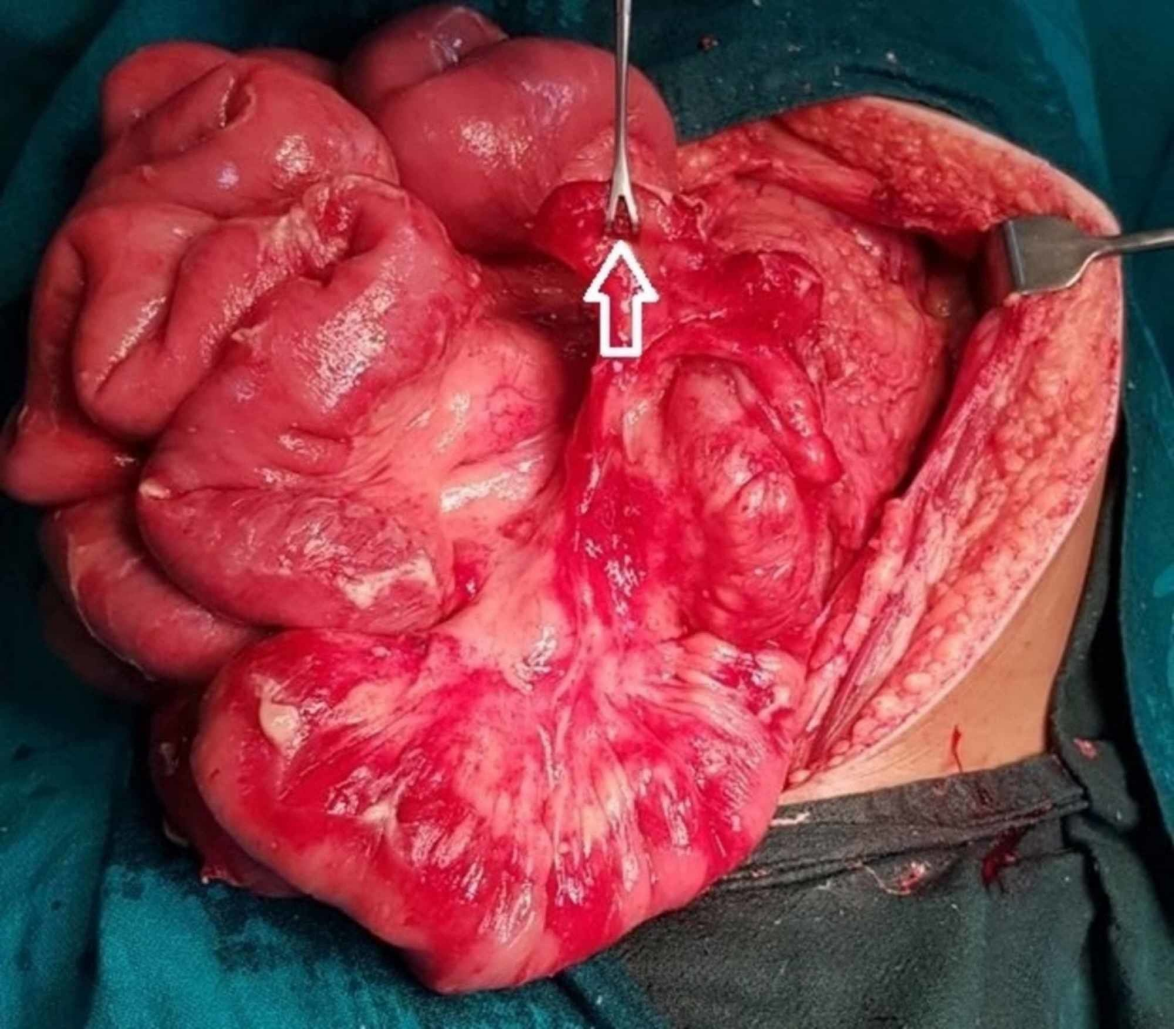
Intraoperative findings showing appendix (unfilled block arrow) on the left side of the abdomen

Due to a lack of surgeon's experience in performing laparoscopic surgery in relatively challenging anatomy and anticipation of bands which may have been missed by imaging, the laparoscopic procedure was not used and surgery via midline incision was planned. Preoperative blood investigations showed leukocytosis (11,800 with 90% neutrophils), normal liver and renal function tests, and positive C-reactive protein. Urinalysis was unremarkable. The chest X-ray showed no abnormalities. The intraoperative findings were inflamed appendix with appendicolith with healthy base arising from the cecum which was fixed in the left lower quadrant with minimal free fluid. The duodenojejunal flexure with a whole small bowel was seen on the right side and the large bowel was seen on the left side (Figure 5). Ladd’s procedure was performed which involved appendectomy, the release of Ladd’s bands along with the realignment of bowel loops to prevent further rotation, and band formation. The postoperative period was uneventful, and the diagnosis was correlated with histopathological examination of the appendix. We discharged him on the fourth postoperative day during which he was in a good physical condition and tolerating his diet. He was doing fine till one month postoperatively. The patient was lost to further follow-up which was later found out to be due to geographical inaccessibility.

## Discussion

A person with acute appendicitis typically presents with vague epigastric or periumbilical pain which later migrates to the right lower quadrant, anorexia, nausea, vomiting, and low-grade fever. While examining patients with such symptoms, we usually come across rigidity, positive psoas sign, fever, and rebound tenderness [5]. These classic findings occur in only about 50% of the patients. There is a high chance of missed or delayed diagnosis when one presents atypically with left lower quadrant pain with the reported rate of misdiagnosis as high as 24% [6].

Differentials of left lower quadrant pain include colitis, diverticulitis, inflammatory bowel disease, intestinal obstruction (including volvulus) or perforation, nephrolithiasis, pyelonephritis, testicular torsion, epididymitis, atypical right-sided appendicitis, left-sided appendicitis, and left-sided primary epiploic appendagitis [7,8].

In the present case, we initially suspected diverticulitis. The underlying intestinal malrotation delayed the correct diagnosis of acute appendicitis. It was only after the CT scan was done malrotation was identified. Two previous case reports have similarly misdiagnosed left-sided acute appendicitis with intestinal malrotation as diverticulitis [9,10]. In the latter case, misdiagnosis led to a delay in definitive management, with the patient subsequently undergoing emergency laparotomy [10]. Thus, an accurate and timely diagnosis is critical to prevent these complications.

Intestinal malrotation occurs when there is either nonrotation or incomplete rotation of the primitive intestinal loop around the SMA axis during the first 10 weeks of fetal life [11]. It is a rare anomaly with an approximate incidence of one in every 500 live births (0.2%; range, 0.03%-0.5%) [12,13]. Most cases (93%) of intestinal malrotation present in the first month of life with bilious vomiting. However, it is uncommon in adults (0.1%-0.5%) and usually remains asymptomatic [14]. Symptomatic adults experience chronic abdominal pain (87%) [15]. Our patient had Stringer type 1a malrotation which is often asymptomatic and is the most common malrotation encountered in adults [11,16].

Acute appendicitis occurs even rarely in association with intestinal malrotation [4,17]. This makes the diagnosis of left-sided acute appendicitis a clinical and radiological challenge. The average age of presentation with acute appendicitis in patients with intestinal malrotation was between 8 and 51 years, which indicates that a person with asymptomatic intestinal malrotation may develop symptoms even in advanced ages [17].

It is imperative to differentiate left-sided acute appendicitis associated with malrotation from that associated with situs inversus totalis, in which every organ, including duodenum, duodenojejunal junction, small and large bowel, cecum, and appendix, is located in a mirror position to situs solitus [17]. In addition to these features, a left-sided liver and right-sided spleen and stomach serve as clues to the correct diagnosis of situs inversus totalis. Chest X-ray is important to obtain at this point for ruling out situs inversus totalis which may be confused with intestinal malrotation. More than two-thirds of the left-sided appendicitis is due to situs inversus totalis rather than intestinal malrotation [4].

USG is considered to be the best initial imaging modality, especially for children. USG may show an inversion of SMA and SMV relationship with a blind noncompressible aperistaltic tube on the left side. CECT of the abdomen may also show an inversion of SMA and SMV, along with large bowel predominantly on the left side, small bowel predominantly on the right side, and concomitant feature of acute appendicitis [18]. CT is useful both in the diagnosis of left-sided acute appendicitis and in detecting associated rotational anomalies and related complications [19].

Ladd's procedure is generally performed when intestinal malrotation presents as a volvulus by an open or laparoscopic approach. However, this procedure becomes optional when malrotation is found incidentally or with other intra-abdominal pathology like acute appendicitis. Laparoscopic appendectomy is considered to be the gold standard of treatment of left-sided appendicitis [20]. But unlike open procedures, laparoscopic procedures have a steep learning curve and are not well established in low resource settings like ours.

## Conclusions

Given the rarity of acute appendicitis associated with intestinal malrotation, an increase in awareness of this anatomical variant is essential among emergency physicians, radiologists, and surgeons for prompt diagnosis and timely intervention. Although the clinical picture may be confusing, imaging modalities greatly help in diagnosing this condition. Appropriate and timely intervention could minimize the risk of detrimental consequences like intestinal perforation/abscess formation and prolonged hospital stay.
